# Mechanism of EHMT2-mediated genomic imprinting associated with Prader-Willi syndrome

**DOI:** 10.21203/rs.3.rs-4530649/v1

**Published:** 2024-07-03

**Authors:** Sung Eun Wang, Yubao Cheng, Jaechul Lim, Mi-Ae Jang, Emily N. Forrest, Yuna Kim, Meaghan Donahue, Sheng-Nan Qiao, Yan Xiong, Jian Jin, Siyuan Wang, Yong-hui Jiang

**Affiliations:** 1 Department of Genetics, Yale University School of Medicine, 333 Cedar St, New Haven, CT 06520, USA; 2 Immunobiology, Yale University School of Medicine, 333 Cedar St, New Haven, CT 06520, USA; 3 College of Veterinary Medicine and Research Institute for Veterinary Science, Seoul National University, Gwanak-ro 1, Gwanak-gu, Seoul 08826, South Korea; 4 Department of Laboratory Medicine and Genetics, Samsung Medical Center, Sungkyunkwan University School of Medicine, Seoul 06351, South Korea; 5 St. Jude Children’s Research Hospital, 262 Danny Thomas Place Memphis, TN 38105, USA; 6 Mount Sinai Center for Therapeutics Discovery, Departments of Pharmacological Sciences, Oncological Sciences and Neuroscience, Tisch Cancer Institute, Icahn School of Medicine at Mount Sinai, New York, NY 10029, USA; 7 Cell Biology, Yale University School of Medicine, 333 Cedar St, New Haven, CT 06520, USA; 8 Neuroscience, Yale University School of Medicine, 333 Cedar St, New Haven, CT 06520, USA; 9 Pediatrics, Yale University School of Medicine, 333 Cedar St, New Haven, CT 06520, USA

## Abstract

Prader-Willi Syndrome (PWS) is caused by loss of expression of paternally expressed genes in the human 15q11.2-q13 imprinting domain. A set of imprinted genes that are active on the paternal but silenced on the maternal chromosome are intricately regulated by a bipartite imprinting center (PWS-IC) located in the PWS imprinting domain. In past work, we discovered that euchromatic histone lysine N-methyltransferase-2 (EHMT2/G9a) inhibitors were capable of un-silencing PWS-associated genes by restoring their expression from the maternal chromosome. Here, in mice lacking the *Ehmt2* gene, we document un-silencing of the imprinted *Snrpn/Snhg14* gene on the maternal chromosome in the late embryonic and postnatal brain. Using PWS and Angelman syndrome patient derived cells with either paternal or maternal deletion of 15q11-q13, we have found that chromatin of maternal PWS-IC is closed and has compact 3D folding confirmation. We further show that a new and distinct noncoding RNA preferentially transcribed from upstream of the PWS-IC interacts with EHMT2 and forms a heterochromatin complex to silence gene expression of *SNRPN* in CIS on maternal chromosome. Taken together, these findings demonstrate that allele-specific recruitment of EHMT2 is required to maintain the maternal imprints. Our findings provide novel mechanistic insights and support a new model for imprinting maintenance of the PWS imprinted domain.

## Introduction

Imprinted genes, unique to mammals and flowering plants, are regulated such that they are expressed from either the maternal or paternal allele but not both^1,2^. Frequently clustered in the same chromosomal domain, imprinted genes are coordinately controlled by an imprinting center (IC) or control region (ICR). Since the discovery of genomic imprinting^3,4^, investigations have focused on two fundamental questions: how cells recognize the paternal and maternal alleles and how cells maintain allele-specific repression of imprinted genes. The imprinting domain of chromosome 15q11.2-q13, implicated in the pathogenesis of both Prader-Willi syndrome (PWS) and Angelman syndrome (AS), has long been an established model for investigating genomic imprinting^5–7^. The 15q11-q13 imprinting domain contains a PWS imprinting center (PWS-IC) and a AS-imprinting center (AS-IC) and two functionally distinct and oppositely imprinted regions^8–10^. Paternally expressed genes *SNURF/SNRPN*, *SNORD116, SNORD115,* and *SNHG14* are located within a 0.5Mb region distal to the PWS-IC and paternally expressed genes *NDN*, *MAGLE2*, and *MKRN3* are ~1.5 Mb proximal to the PWS-IC. However, how the PWS-IC controls allele specific expression bidirectionally remains poorly understood.

The genomic organization of the 15q11.2-q13 imprinting domain is highly conserved in the chromosome 7C region in mice^11^ and the general imprinting regulation mechanism is highly similar in humans and rodents. However, at a sequence level, specific regulatory elements are not highly conserved, presenting challenges for mechanistic analyses in animal models. At a structural level, the critical region of PWS-IC has been mapped to a 4.1 kb region that includes the CpG island (CGI) encompassing exon 1 of *SNRPN*^8,9,12^. PWS-IC is methylated (5mC) on the maternal chromosome in oocytes but unmethylated on the paternal chromosome in sperm^13^. Thus, after fertilization, the maternal methylation signature provides an allele-specific methylation imprint that becomes resistant to epigenetic reprogramming^14^. Although the maternal methylation signatures correlate with repressed expression of paternally expressed genes on the maternal chromosome, exactly how the methylated PWS-IC silences these genes has remained elusive.

The PWS-IC on the paternal chromosome regulates the expression of paternally expressed genes^8,15^, and when deleted from the paternal chromosome in germ cells in both human and mice, the unmethylated pattern of the paternal chromosome became methylated pattern that resemble of maternal chromosome and the paternal gene expression is lost in offspring^15–18^. In contrast, the role of PWS-IC on the maternal chromosome and the mechanism underlying silenced expression in the maternal chromosome are unknown. In mouse embryonic stem cells (mESCs) and embryos null for the DNA methyltransferase *Dnmt1*, methylation of CGI in the PWS-IC on the maternal chromosome was lost^19,20^. Although transcription of the paternally expressed *Snrpn* gene on the maternal chromosome remained repressed in *Dnmt1*-null ES cells^19^, *Snrpn* gene expression was unsilenced in *Dnmt1*-null embryos^20^. Similarly, treatment with DNA methylation inhibitors in PWS patient-derived cells with a paternal 15q11.2-q13 deletion reduced CGI DNA methylation (5mC) and unsilenced *SNRPN* expression from the maternal chromosome^21,22^. In our large-scale small molecule screen in *Snrpn-EGFP* mouse embryonic fibroblasts, we discovered that inhibitors of the nuclear histone lysine methyltransferase EHMT2, also known as G9a, were capable of unsilencing the imprinted *SNRPN* and *SNHG14* genes from the maternal chromosome, both in human fibroblasts derived from patients with PWS and in a PWS mouse model^21^. Unexpectedly, EHMT2 inhibitors unsilenced the repressed expression but did not change the DNA methylation of PWS-IC^21^.

EHMT2 catalyzes mono- and di-methylation of histone H3 lysine 9 (H3K9) or other non-histone proteins^23^. H3K9me2 is a repressive mark for epigenetic transcriptional silencing leading to heterochromatin assembly with chromodomain-containing proteins of the HP 1 family^23–26^. EHMT2 plays a significant role to maintain imprinted DNA methylation, including at the PWS-IC, in embryonic stem cells; this activity is independent from its catalytic activity responsible for H3K9 methylation^27^. Recent chromatin structure studies support an important role for EHMT2-mediated H3K9 in the 3D genome organization of chromatin^28^. Consistently, chromatin loops and topological associated domains (TADs) undergo alterations in EHMT2-deficient mouse ESCs or EHMT2 inhibitor-treated hepatocytes, resulting in de-repression of a subset of nuclear envelope-bound genes^29–31^.

Here, we set out to decipher how EHMT2 inhibition instructs unsilencing of PWS imprinted genes. We found that *Ehmt2* deficiency in mouse forebrain is sufficient to unsilence the expression of imprinted *Snrpn* on the maternal chromosome, without any reduction in DNA methylation of PWS-IC. In human PWS and AS patient derived fibroblasts with paternal or maternal deletion of 15q11.2-q13 respectively, we found that EHMT2 preferentially binds to maternal PWS-IC and enriches H3K9me2, independent of the allele-specific DNA methylation status at the PWS-IC, and that chromatin of the maternal PWS imprinting domain is in a closed and compact 3D folding confirmation. We further show that maternal noncoding RNA transcribed upstream of the PWS-IC interacts with EHMT2 and forms a heterochromatin complex to silence gene expression of *SNRPN* in CIS on maternal chromosome. These fundamental findings support that EHMT2 plays a central role of maintaining the silenced expression of paternally expressed genes in the maternal chromosome.

## Results

### *Ehmt2* deficiency unsilenced the expression of imprinted *Snprn* gene from the maternal chromosome

**Extended Data Fig. 1:**
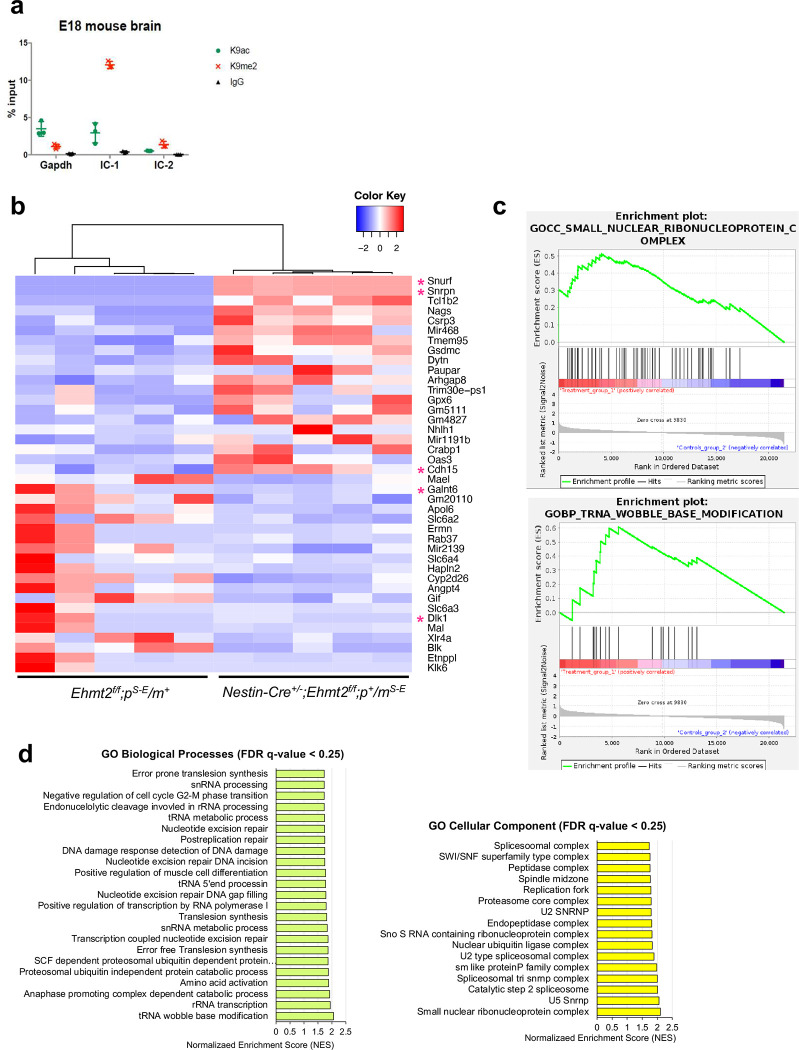
(**a**) ChIP–qPCR quantification of H3K9me2 and H3K9ac in brains of E18 mice at basal condition (n=3 per group). (**b**) Heat map of differentially expressed genes in the comparison of control (*Ehmt2*^*f/f*^;*m*^+^*/p*^*Snrpn-EGFP*^) and Ehmt2 cKO (*Nestin-Cre*^+^*/+*;*Ehmt2*^*f/f*^;*m*^*Snrpn-EGFP*^*/p*^+^*)* forebrain at p10. (**c**) Enrichment plot for top one data set enriched in gene ontology analysis shows the profile of the running ES Score and positions of gene set members on the rank-ordered list. (**d**) Gene sets significantly enriched (FDR q-val < 0.25) in the *Nestin-Cre*^+^*/+;Ehmt2*^*f/f*^*;m*^*Snrpn-EGFP*^*/p*^+^ using the Gene Ontology Cellular Components (left) and Biological Process (right), order by NES with the number of genes assigned to each gene set.

**Figure 1.**
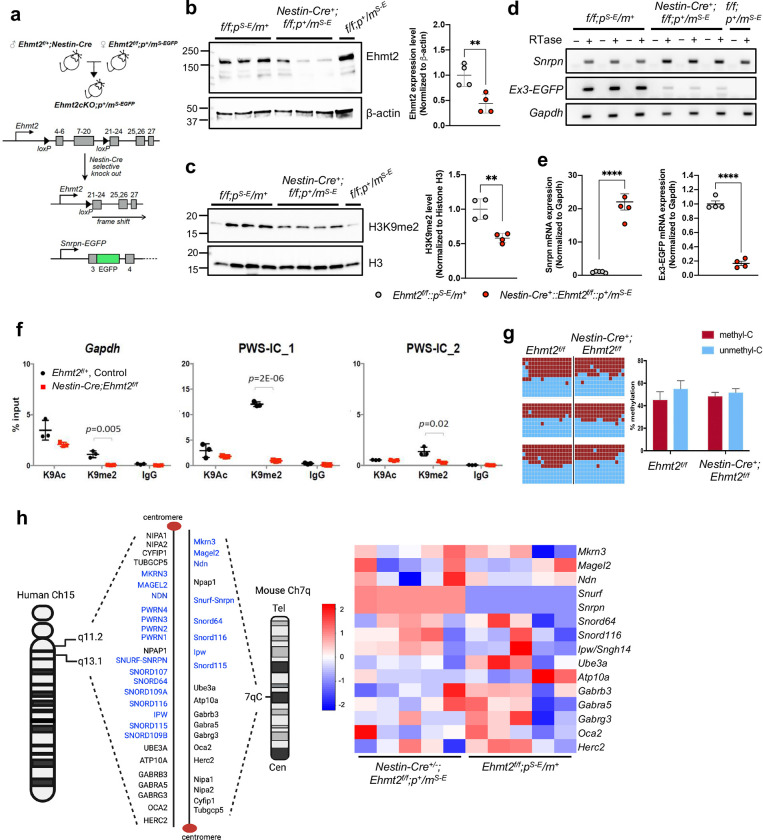
Nestin-Cre mediated loss of *Ehmt2* in forebrain is sufficient to unsilenced the imprinted *Snrpn* gene in the maternal chromosome. **(a)** Schematic figure shows a strategy for generating *Ehmt2* cKO mice with maternal *Snrpn-EGFP* (Exon 3 of *Snrpn* is fused in-frame with EGFP, Ex3-EGFP) **(b)** The level of EHMT2 protein was significantly decreased in forebrains of *Ehmt2* cKO mice at p10. **(c)** H3K9me2 level was decreased in forebrains of *Ehmt2* cKO mice. **(d)** RT–PCR analysis detected the expression of *Snrpn-EGFP* (Ex3-EGFP) in the forebrains of *Ehmt2* cKO mice carrying maternal *Snrpn-EGFP* (RTase: +/−, with or without reverse transcriptase). (**e**) quantitative RT–qPCR analysis of *Snrpn* and *Snrpn*-EGFP mRNA levels in the forebrains. (**f**) ChIP–qPCR quantification of H3K9me2 and H3K9ac in brains of *Ehmt2* cKO mice. Enrichment of H3K9me2 was significantly reduced PWS-IC (IC-1 and IC2) of *Ehmt2* cKO mice. **(g)** Comparison of the DNA methylation in PWS-IC between control and *Ehmt2* cKO mice. (**h**) The genes in human 15q11.2-q13 and its homologous region mouse central chromosome 7. The paternally expressed genes are highlighted in blue. Heat map of the expression of genes located in the central chromosome 7 between control (*Ehmt2*^*f/f*^*;m*^+^*/p*^*Snrpn-EGFP*^) and *Ehmt2* cKO (*Nestin-Cre*^+^*/+;Ehmt2*^*f/f*^*;m*^*Snrpn-EGFP*^*/p*^+^) forebrain at p10 (adj p-value < 0.05).

Based on our previous EHMT2/G9a inhibitor study^21^, we hypothesized that expression of imprinted PWS genes from the maternal chromosome is unsilenced upon EHMT2 depletion. Because *Ehmt2* deficiency in germline results in early embryonic lethality before E9.5 day^32^, we bred *Ehmt2* flox/flox mice^33^ with *Nestin-Cre* mice to generate a brain-specific *Ehmt2* knockout starting embryonic day 11 on the maternal *Snrpn-EGFP* reporter background (*Nestin-Cre*^+/−^; *Ehmt2*^*f/f*^; *m*^*Snrpn-EGFP*^/*p*^+^) (Fig. 1a). We then confirmed that EHMT2 protein levels and H3K9me2 levels were reduced in p10 *Ehmt2* conditional knockout mouse forebrain tissue (Fig. 1b–c) and that unsilencing of *Snrpn-EGFP* from the maternal chromosome was readily detected by RT-PCR (Fig. 1d–e). Through ChIP analysis, we validated that H3K9me2 levels on the PWS-IC were significantly decreased in *Ehmt2* deficient brains (Fig. 1f; Extended data Fig. 1a) but that CGI DNA methylation was not affected (Fig. 1g). Through RNA-seq analysis, we documented overexpression of *Snurf-Snrpn* (a bicistronic transcript) in *Ehmt2* deficient brains (Fig. 1h). We noted the increased expression of *Ipw/Snhg14* and *Snord116* which is the part of extended transcript of *Snurf/Snrpn* was less consistent, and this is likely due to the low sensitivity of bulk RNA-seq for non-coding RNAs containing low copy repeat. Notably, the expression of other PWS-associated paternally expressed genes of *Magel2*, *Ndn*, and *Mkrn3* in distal side of PWS-IC in chromosome 7C region was not consistently upregulated. This observation indicates for the first time that silencing of paternally expressed genes at distal and proximal side is mediated by a different mechanism. We were intrigued about the finding that other paternally expressed genes such as *Cdh15*, *Galnt6*, and *Dlk1* were either significantly upregulated (*Cdh15*) or downregulated (*Galnt6* and *Dlk1*) (Extended Data Fig. 1b). This suggests that EHMT2 may act as both repressor and activator in regulating imprinted genes. Furthermore, when conducting gene ontology analysis, we found that genes differentially regulated in the forebrain of *Ehmt2* deficient mice were significantly enriched for RNA splicing and DNA repair process (Extended data Fig. 1c, 1d). Taken together, these findings demonstrate that EHMT2 rather than DNA methylation in PWS-IC is associated with silencing of paternally expressed genes *Snurf-Snrpn* at the distal side but not *Ndn*, *Magel2*, and *Mkrn3* at the proximal side in the maternal chromosome.

### EHMT2 binds preferentially to the PWS-IS of maternal chromosome that is independent from DNA methylation

**Extended Data Fig. 2:**
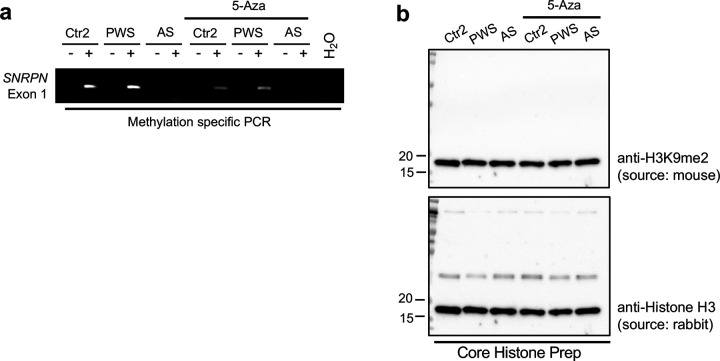
(**a**) Methylation of CGI of PWS-IC overlapped with SNRPN Exon 1 was decreased after treatment with 5-Aza. (**b**) Representative western blot images for H3K9me2 and Histone H3. The level of H3K9me2 was not decreased in human fibroblasts after treatment with 5-Aza (Control, PWS; Prader-Willi Syndrome, AS; Angelman syndrome).

**Figure 2.**
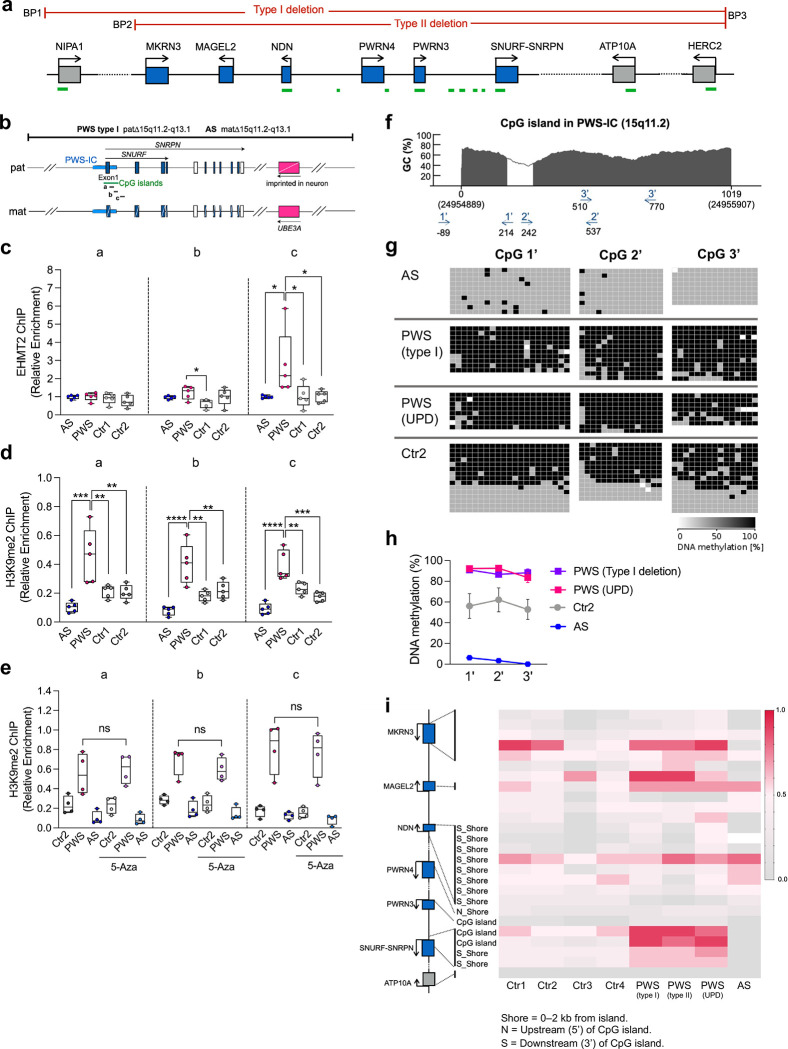
Maternal allele-specific enrichment of EHMT2 at PWS-IC that is independent on DNA methylation. **(a)** Schematic diagram of PWS-associated imprinted domain on chromosome 15 (blue allele; paternally expressed genes, gray allele; biallelic expressed gene, BP; break point found in PWS patient with 15q11.2-q13 deletion. Green bar; CGIs). **(b)** Schematic diagram of primer binding region (a,b,c within PWS imprinting center. **(c-e)** ChIP–qPCR quantification of EHMT2 and H3K9me2 on PWS-IC in human fibroblasts derived from Angelman syndrome (AS) and PWS patients (AS; paternal 15q11.2-q13 deletion, PWS; maternal 15q11.2-q13 deletion, Ctr; Control). H3K9me2 is significantly abundant on PWS-IC of maternal chromosome compared to AS and Control after treatment of 5-Aza as a DNMT1 inhibitor. **(f)** Schematic diagram of primer binding site on CGI in PWS-IC for bisulfite genomic sequencing. **(g)** Comparison of DNA methylation in AS (paternal CGI), PWS type I deletion and Uniparental disomy (UPD) (maternal CGI), and a control (gray; unmethylated CpG, black; methylated CpG). **(h)** Quantification of DNA methylation level on PWS-IC. **(i)** Genome-wide methylation analysis shows that allele-specific DNA methylation is shown in PWS-IC but not other CGIs. Heat map depicts average methylation scores (0; unmethylated CpG, 1;methylated CpG).

To examine interactions between EHMT2 and the maternal and paternal alleles of the PWS-IC, we performed ChIP analysis in human fibroblasts derived from a patient with PWS (a 6Mb paternal deletion of 15q11.2-q13) and a patient with AS (a 6 Mb maternal deletion of 15q11.2-q13) (Fig. 2a, 2b). Compared to the paternal chromosome, we found that EHMT2 binding and H3K9me2 levels were significantly enriched in the PWS-IC of the maternal chromosome (Fig. 2c–d). To investigate whether methylation of CpGs of PWS-IC is a prerequisite for H3K9me2 enrichment, we treated fibroblasts with DNA methylation inhibitor of 5-Aza-2’-deoxycytidine (5-Aza) and confirmed by bisulfite-based PCR that CpG methylation (5mC) on PWS-IC including CGI and exon 1 of the *SNRPN* gene was reduced (Extended Data Fig. 2a). Because H3K9me2 enrichment in the PWS-IC was not altered despite the reduction of DNA methylation, we conclude that EHMT binding and H3K9me2 enrichment in the PWS-IC on the maternal chromosome does not require CpG methylation (Fig. 2e, Extended Data Fig. 2b).

We further investigated the methylation status of CpG dinucleotides in the 15q11.2-q13 imprinting domain in PWS patient fibroblasts with a paternal deletion or maternal uniparental disomy (UPD) and in AS patient with a maternal deletion (Fig. 2f–g). Using bisulfite genomic sequencing, we confirmed that maternal allele-specific DNA methylation for the CGI was associated with the PWS-IC and noted that, relative to the distal region (CpG 3’), CG dinucleotides in the proximal CGI (CpG 1’) region were more methylated in paternal chromosomes (Fig. 2h). We then used methylome arrays to extend the methylation analysis to the remaining 15q11.2-q13 regions. This analysis confirmed allelic methylation of CGI of *SNRPN* but unexpectedly, did not reveal consistent allele-specific methylation of CGIs associated with other paternally expressed genes, such as previously reported for *MAGEL2*, *NDN*, and *MKRN3* using traditional low throughput methods^34–36^ (Fig. 2i). The discrepancy may reflect the inter-individual variability of methylation status or different resolution of method used for DNA methylation analyses.

### Allele-specific chromatin accessibility is associated with PWS imprinting domain but not affected by EHMT2 inhibition

**Figure 3.**
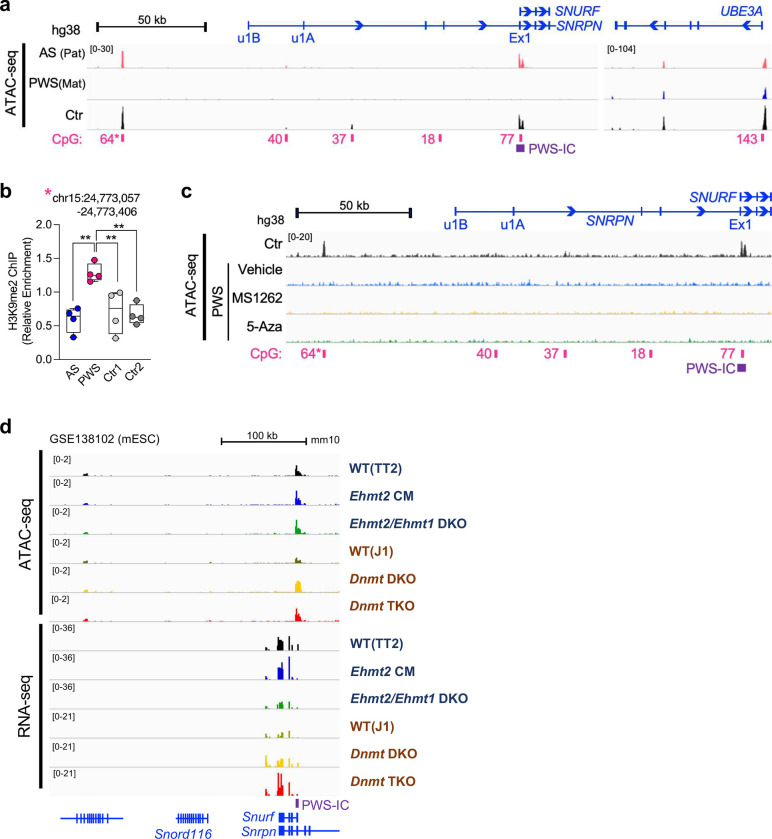
Allele-specific chromatin state of 15q11.2-q13 region that was not affected by EHMT2 and DNA methylation inhibitors. **(a)** Genome viewer screenshot of ATAC-seq analysis illustrating a closed chromatin state of maternal imprinting domains (PWS) and an open chromatin state of paternal imprinting domains (AS). The control (Ctr) also shows an open chromatin state. **(b)** ChIP–qPCR quantification of H3K9me2 on the upstream region of *SNRPN* gene (§, ATAC-seq peak) in human fibroblasts derived from Angelman syndrome (AS) and PWS patients with a 15q11.2-q13 deletion. **(c)** Genome viewer screenshot illustrating maternal imprinting domains (PWS) remain to be a closed chromatin after treatment of MS1262 (EHMT2 inhibitor) or 5-Aza (DNMT1 inhibitor). **(d)** Genome viewer screenshot demonstrating *Ehmt2* catalytic mutant (CM) or *Ehmt2/Ehmt1* double knockout (DKO) not contributing to open chromatin status on PWS-IC in mouse embryonic stem cells (ESC).

Next, to examine chromatin accessibility in the PWS-associated region, we performed ATAC-seq using PWS and AS fibroblasts with a paternal or maternal deletion. This analysis revealed that regulatory regions, including the CGI in the PWS-associated region on maternal chromosome (PWS), were in a closed state compared to the paternal chromosome (AS) and normal control (Ctr) (Fig. 3a). The peaks of chromatin accessibility in the CGIs were similar between control and AS, suggesting that the paternal region was in an open state to maintain active transcription of PWS-associated genes. The regulatory *UBE3A* regions that are bi-allelically expressed in fibroblasts were in an open chromatin state in both maternal and paternal chromosomes (Fig. 3a). We noted a significant peak of chromatin accessibility at the upstream region of *SNRPN* (50 kb from u1B) that was associated with the paternal chromosome (64 CpGs, marked with * in Fig. 3a). ChIP analysis revealed that this region was more enriched with H3K9me2 on the maternal chromosome than the paternal chromosome (Fig. 3b).

**Extended Data Fig. 3:**
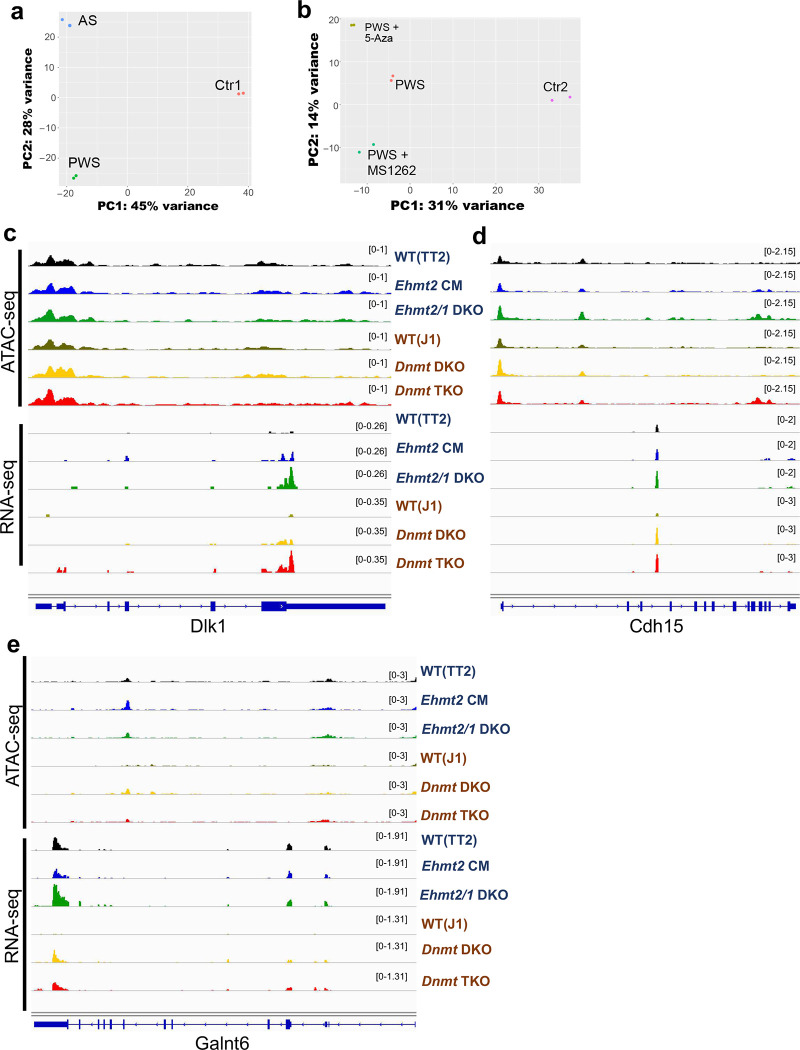
(**a, b**) Principal Component Analysis (PCA) assay showing the separate clustering of different samples and the clustering of each replicate of the same condition together. (**c-e**)Genome viewer screenshot demonstrating *Ehmt2* catalytic mutant (CM), *Ehmt2/Ehmt1* double knockout (DKO), *Dnmt3a/3b* (DKO), or *Dnmt1/3a/3b* triple knockout (TKO) contributing to overexpression of imprinted genes, (**c**) *Dlk1*, (**d**) *Cdh15*, (**e**) *Galnt6* regardless of open/closed chromatin status in mouse embryonic stem cells.

To determine whether EHMT2 or DNMT1 inhibitors change the chromatin states of the PWS imprinting domain on the maternal chromosome, we treated PWS fibroblasts with a paternal deletion with MS1262^37,38^, a new EHMT2 inhibitor, or 5-Aza, a known DNMT1 inhibitor. We confirmed that both MS1262 and 5-Aza treatments led to the reactivation of the *SNPRN* gene from the repressed maternal chromosome. Through the ATAC-seq analysis, we did not revealed that these treatments resulted in a more open chromatin state of PWS imprinting domain despite the observed unsilencing of paternally expressed gene from the maternal chromosome (Fig. 3c), and despite Principal Component Analysis (PCA) supporting that treatment with DNMT1and EMHT2 inhibitors resulted in significant gene expression differences in PWS fibroblasts (Extended Data Fig. 3a–3b). In line with this finding, increased chromatin accessibility in PWS-IC was not observed in *Ehmt2* catalytic mutant (CM) or *Ehmt2/Ehmt1* double knockout (DKO) mouse ESCs (Fig. 3d). In contrast, the gain of chromatin accessibility along with overexpression of *Snurf-Snrpn* was observed in mouse *Dnmt1/3a/3b* triple KO (TKO) ESCs (Fig. 3d). The paternally expressed genes of *Dlk1, Cdh15*, and *Galnt6* did not show distinguishable chromatin state changes in *Ehmt2/1* deficient or *Dnmt1/3a/3b* TKO mouse ESCs (Extended Data Fig. 3c–3e) even though their expression was upregulated. These results indicate that chromatin accessibility change may not be essential to enable gene expression of repressed imprinted PWS alleles and that instead other regulatory factors inducing spatiotemporal chromatin architecture may be considered as suggested in recent work^39–41^.

### Chromatin organization of PWS imprinting domain shows allele-specific chromatin conformation

**Extended Data Fig. 4:**
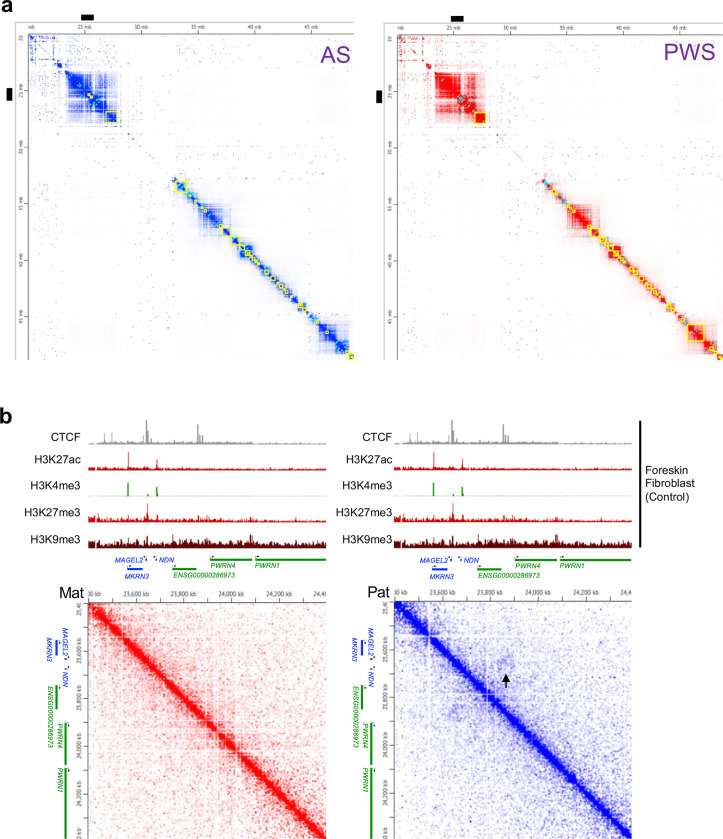
(**a**) 2D contact map of a region of chromosome 15 showing TADs (yellow) as boxes of enriched contact frequency and loops (cyan) at 50-Kb resolution. A region of chr15:24,500,000–26,200,000 (Fig. 4a) is indicated by black bar in 2D contact map. (**b**) This Juicebox screenshot visualizing a region of 1.1 Mb locus (chr15:23,400,000–24,400,000) at 5-Kb resolution (Normalization; SCALE). A paternal specific loop is indicated by arrow (black) in 2D contact map.

**Figure 4.**
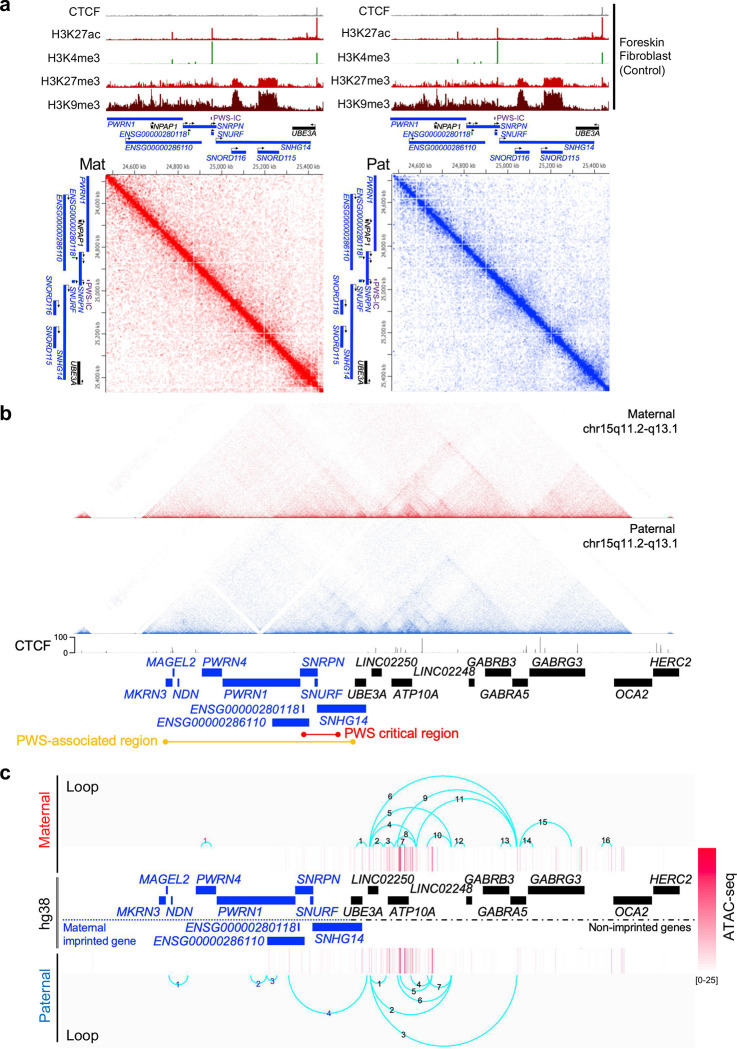
Allele-specific chromatin conformation shows in PWS imprinting domains by Hi-C analysis. **(a)** This Juicebox screenshot visualizing a region of 1.7 Mbp locus (chr15:24,500,000–26,200,000, hg38) at 5-Kb resolution. (Normalization; SCALE) **(b)** Wash U Epigenome Browser snapshot showing CTCF (control) Hi-C track of a 5.5 Mb region of human chromosome 15q11.2-q13 at 500 bp resolution normalized using SCALE. The triangle shapes in the Hi-C track depict chromatin domains in human fibroblasts derived from PWS and AS patients (The color scale of the heatmap; higher contact counts corresponding to a darker color). **(c)** Genome viewer screenshot illustrating 1D representation of the DNA fragment that forms the loop in PWS-associated imprinted domains of maternal or paternal chromosome, partially matched with ATAC-seq peaks. (*UBE3A*; a paternal and neuron cell type specific imprinted gene in brain).

Next, to determine physical chromatin interactions in maternal or paternal chromosome, we performed Hi-C analysis of human fibroblasts derived from patients with a large 15q.11-q13 deletion on the paternal chromosome (PWS) or maternal chromosome (AS). Each Hi-C contact matrix was aligned with reference tracks of CTCF and histone marks contributing to the overall chromatin structure (Fig. 4a). In line with a previous report^42^, there are no strong TAD boundaries in the PWS-critical region including the PWS-IC (Fig. 4b, Extended Data Fig. 4a), consistent with the scarcity of CTCF binding in this region that is in contrast with other imprinting domains such as *H19-IGF2* in chromosome 11p15 region^42^ (Fig. 4a). We observed maternal and paternal specific chromatin loops in imprinting loci, with significantly more loops in paternal than maternal loci (Fig. 4c). Biallelic loci showed more loops and open chromatin peaks than imprinted loci (Fig. 4c). Interestingly, imprinted genes located > 1Mb upstream from the PWS-IC showed strong CTCF peaks with a paternal-specific loop (Extended Data Fig. 4b). Taken together, these results indicate an allele-specific chromatin conformation of PWS imprinting domain and suggest a role of chromatin conformation in regulating the imprinting maintenance to silence gene expression on the maternal chromosome.

### Chromatin tracing reveals allele-specific 3D folding organization of PWS imprinting domains in the human fibroblasts

**Figure 5.**
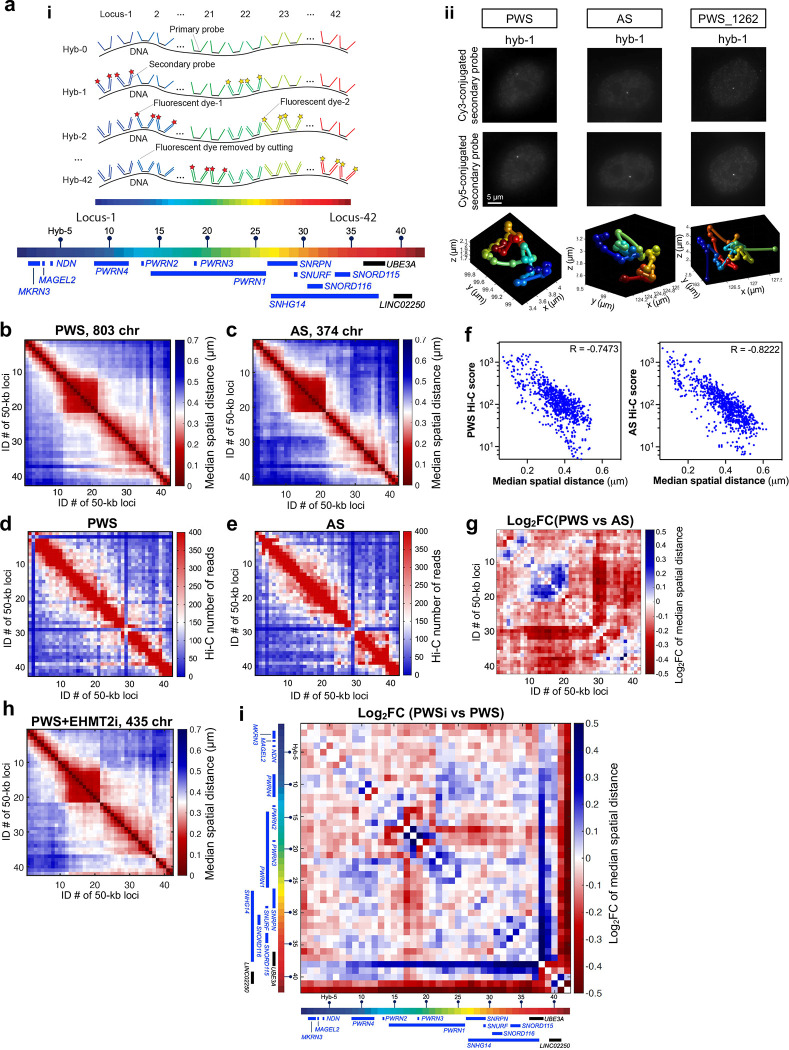
Chromatin tracing uncovers the 3D conformation of PWS regulatory region (Target region: Chr15:23,500,000–25,650,000, hg38). **(a)** (i) Schematic illustration of the chromatin tracing strategy. (ii) Representative images showing foci corresponding to maternal/paternal chromosome, with representative chromatin trace, respectively. **(b, c)** Median spatial distance matrix of the traced genomic region (42 consecutive 50-kb loci) in human fibroblasts. **(d, e)** Hi-C contact frequency matrix of the same genomic region as in **b** and **c**. **(f)** Comparison of median inter-loci spatial distance from chromatin tracing with contact frequency measured by Hi-C. **(g)** Log2 fold change of inter-loci distance of PWS versus AS. (**h**) Median spatial distance matrix of the traced genomic region (42 consecutive 50-kb loci) in PWS fibroblasts treated with EHMT2 inhibitor. **(i)** Log2 fold change of inter-loci distance of PWS fibroblasts treated with EHMT2 inhibitor versus control PWS.

To further characterize the chromatin 3D folding architecture, we applied a chromatin tracing method based on multiplexed DNA fluorescence in situ hybridization (FISH)^43,44^ in the human PWS and AS fibroblasts with a paternal or maternal deletion. We partitioned the critical PWS imprinting domain into 42 consecutive 50-kb segments, spanning the 2.1-Mb genomic region from genome coordinates of chr15:23,500,000 to 256,500,000 (hg38), and labeled each segment with 500 unique primary oligonucleotide probes (Fig. 5a). Then, through sequential hybridization, we generated matrices of median spatial distance between each pair of targeted segments for the PWS-associated region on paternal or maternal chromosomes (Fig. 5b, 5c). To validate the chromatin traces, we compared distances with the corresponding contact frequencies from our Hi-C analysis (Fig. 5d, 5e). We found that the inter-loci median spatial distances of paternal or maternal region of interest were highly correlated with the Hi-C contact frequencies, with correlation coefficients of −0.7473 and −0.8222 for paternal and maternal region, respectively (Fig. 5f). To compare chromatin compaction between maternal and paternal regions, we calculated the log2-fold change of median spatial distances between PWS and AS (Fig. 5g). The traced region, which is largely maternally silenced, is overall more compactly folded (smaller distances) in PWS cells than in AS cells, whereas a sub-region containing *PWRN4–1* was more compacted in AS cells than in PWS cells. To investigate whether EHMT2 maintains the chromatin conformation of the imprinting domain in the PWS critical region (chr15:24,950 kb-25,300 kb, Hyb #26-#32), we treated PWS fibroblasts with a paternal deletion with the EHMT2 inhibitor (Fig. 5h). When we calculated the log2 fold-change of the median spatial distance between PWS treated with EHMT2 inhibitor and control (Fig. 5i), we did not detect a significant systematic change in 2.1 Mb PWS imprinting domain. However, it should be noted that the interpretation is limited by the 50kb genomic resolution for the probe design and the micro-change of chromatin structure may be missed.

### New maternal noncoding RNAs upstream of PWS-IC recruit EHMT2 and interact with PWS-IC on the maternal chromosome

**Extended Data Fig. 5:**
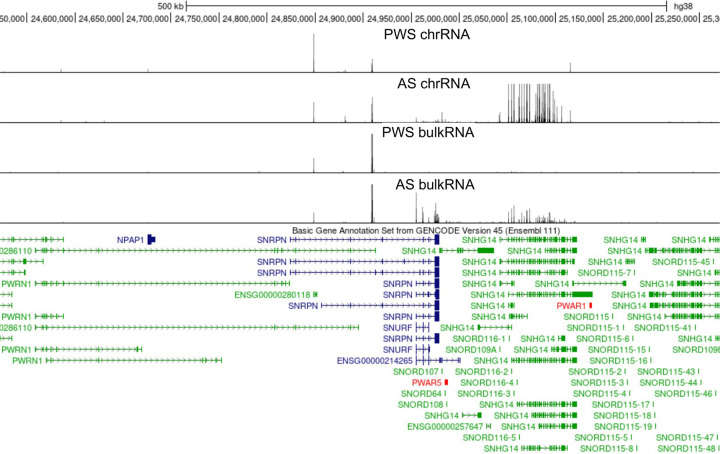
Peaks from chromatin-associated RNA sequencing in the region of chr15:24,550,000–25,250,000.

**Extended Data Fig. 6:**
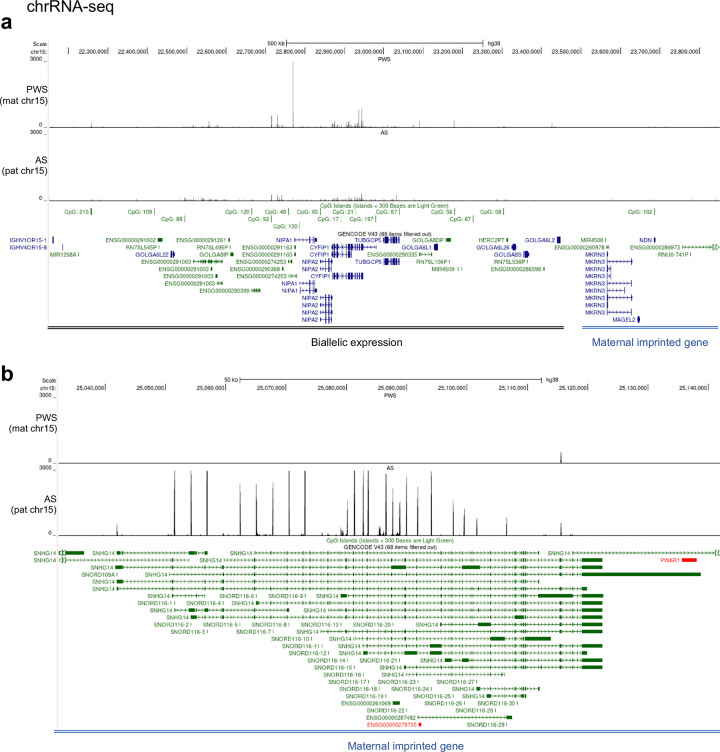
Peaks from chromatin-associated RNA sequencing in the region of (**a**) chr15:22,200,000–23,800,000 and (**b**) chr15:25,040,000–25,140,000.

**Figure 6.**
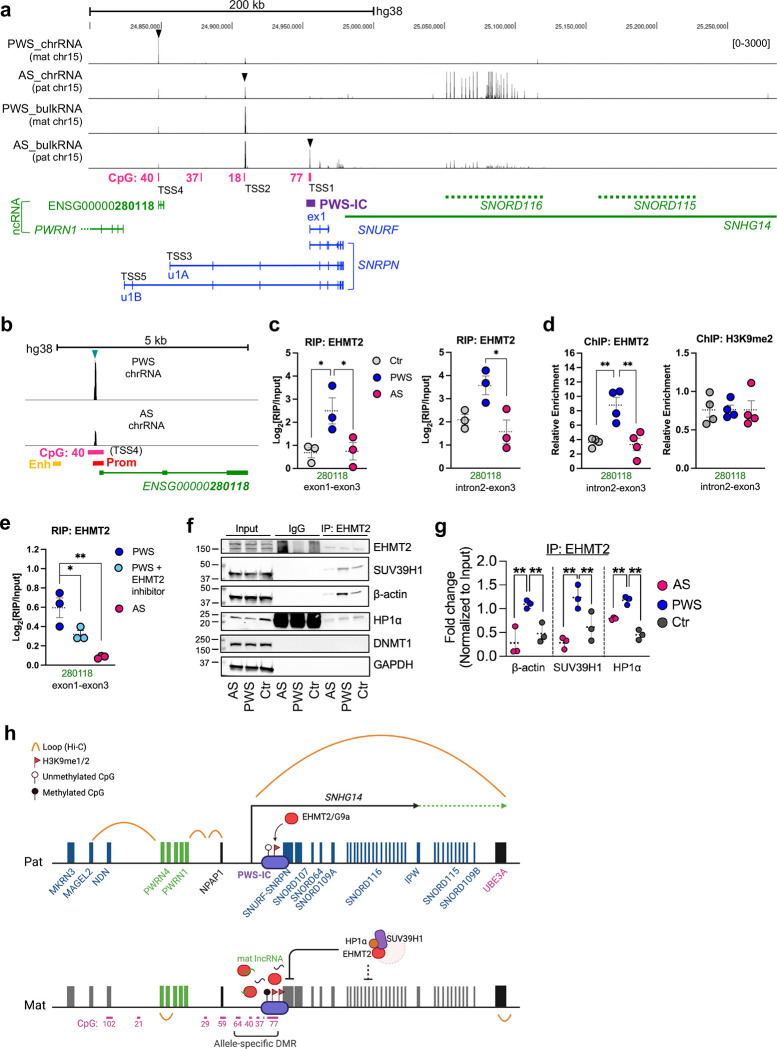
Maternal non-coding RNAs recruit EHMT2 to PWS-IC to silence the expression of *SNRPN/SNHG14* in the maternal chromosome. **(a)** Genome browser screenshot illustrating distribution of chrRNA-seq and bulk RNA-seq reads of ncRNA transcripts upstream of PWS-C in human fibroblasts derived from PWS and AS patients. The transcription start site close to the codding exon 1 of *SNRPN* is defined as a canonical transcription start site 1(TSS1). u1A (TSS3) and u1B (TSS5) are two oocyte specific transcript start sites previously reported^45^. TSS2 and TSS4 that correspond to CGI-18 and CGI-40 are identified from this study. TSS4 is preferentially maternal as shown the expression peak in significantly higher in PWS chrRNA than in AS ChrRNA. (**b)** Genome browser screenshot showing chrRNA-seq reads TSS4 of ncRNA of 280118 in human fibroblasts derived from PWS and AS patients. **(c)** Native RNA-IP (RIP) with EHMT2 antibody showed the interaction between EMHT2 and maternal ncRNAs of using the primers derived from 280118. **(d)** Allele-specific ChIP-qPCR with EHMT2 and H3K9me2 in genomic loci associated with ncRNA transcript 280118 in human fibroblasts. **(e)** RIP-qPCR with EHMT2 antibody after treatment with EHMT2 inhibitor (MS1262) in human fibroblasts. **(f)** Representative blots showing components of EHMT2 repressive complex following immunoprecipitation (IP) of lysates from human fibroblasts with Ab against EHMT2. **(g)** Quantification of co-IP result. Protein levels were normalized to inputs. EHMT2 repressive complexes were more abundant in PWS fibroblasts compared to AS fibroblasts or Ctr. **(h)** Schematic illustration shows a new model of EHMT2-mediated maternal imprinting maintenance based on the data in this study (blue allele: maternal imprinted gene, green allele: maternally imprinted noncoding RNA, NPAP1: monoallelic (paternal) expression in fetal brain, biallelic expression in adult testis and brain, *UBE3A*: neuronal cell type specific paternal imprinted gene, green ncRNA; maternal specific transcription, black ncRNA; biallelic transcription).

Our findings that the DNA methylation at PWS-IC is not essential for maternal allele specific EHMT2 binding in fibroblasts suggest an alternative mechanism to be tested. We then hypothesized that EHMT2 is recruited to PWS-IC by non-coding RNAs to maintain silencing on the PWS imprinting domain on the maternal chromosome. Previous reports delineated three transcript start sites (TSS) of *SNRPN* in humans that include canonical site from exon 1 of *SNRPN* (TSS1, which is paternal-specific), upstream u1A (TSS3) and u1B (TSS5) (Fig. 6a)^45^. The same structure of exon 1 of *Snrpn*, *U1*, and *U2* is also described in mice. *U1* and *U2* are known as oocyte-specific^11^. Four CGIs (40, 37, 18, 77) are mapped within this region, of which the CGI-77 overlaps with PWS-IC and TSS1 whereas TSS2 and TSS3 are not associated with any CGIs. In silico analysis revealed the presence of *PWRN1* and new non-coding RNA (ncRNAs) of ENSG00000280118 (280118) upstream of TSS1(Extended data Fig. 5). *PWRN1* is a previously reported non-coding RNA that displays an isoform and paternal specific in fetal brain but biallelic expression in kidney and testis^46^. 280118 is a transcript (3899bp) with 3 exons that does not overlap with any exon of *PWRN1* and untranslated exons of coding *SNRPN* transcripts. Using bulk RNA-seq, we examined allele specific expression of transcripts upstream of TSS1 in PWS and AS fibroblasts with a paternal or maternal deletion of 15q11.2–13. We found several transcript peaks that overlap with CGI and were either predominantly paternal, maternal, or biallelically expressed (Fig. 6a). As expected, non-imprinted gene loci were transcribed from both paternal and maternal chromosomes in fibroblasts (Extended Data Fig. 6). The in silico analysis predicted possible new TSSs associated with these non-coding RNA transcript peaks. We delineate these new upstream non-coding transcripts as TSS2-TSS5 as diagramed (Fig. 6a). The TSS4 peak associated with transcripts ENSG00000280118 and CGI-40 is predominantly maternal specific by chromatin-associated RNA-seq (chrRNA-seq) (Fig. 6b)^47^. Through RNA immunoprecipitation (RIP) with a EHMT2 antibody, we were able to detect binding between these maternal ncRNAs and EMHT2 (Fig. 6c). We also captured EHMT2 binding to the genomic region encoding the ncRNA (280118) by ChIP-qPCR, but without any change in H3K9me2 level (Fig. 6d). These results indicated that ncRNAs 280118 may recruit EHMT2. We next examined whether EHMT2 inhibition affected the interaction of EHMT2 with ncRNAs in cultured fibroblasts. After EHMT2 inhibitor treatment, which unsilenced expression of *SNRPN* from the maternal chromosome^38^, the interaction between ncRNA and EHMT2 were significantly reduced (Fig. 6e). These results suggest that binding of ncRNA and EHMT2 contributes to the silencing of *SNRPN* gene on the maternal chromosome. EHMT2 is reported to interact with other chromatin regulators such as SUV39H1, heterochromatin proteins of (HP)-alpha (HP1α) and β-actin^24,48–50^. We performed co-immunoprecipitation analysis that confirmed the interaction of EHMT2 with SUV39H1, HP1α, and β-actin (Fig. 6f). Accordingly, EHMT2 protein complex with SUV39H1, HP1α, and β-actin were significantly more enriched in the maternal imprinted domain of 15q11.2-q13 compared to the paternal chromosome of the same region (Fig. 6g). Taken together, our analyses suggest that maternal ncRNAs transcribed from the upstream region of PWS-IC recruit EHMT2 and form a heterochromatin repressor complex to PWS-IC that instruct silencing of imprinted genes in the maternal chromosome.

## Discussion

Here, we deploy comprehensive molecular and high resolution chromatin analyses using PWS and AS patient-derived fibroblast with paternal or maternal deletion respectively to significantly advance our understanding of mechanisms underlying imprinting regulation in 15q11.2-q13 region. Firstly, we show that EHMT2-mediated H3K9me2 but not DNA methylation on the maternal PWS-IC is essential for maintaining the silencing of paternally expressed genes in the maternal chromosome. Secondly, distinct mechanisms operate to silence the expression of paternally expressed genes proximal and distal to the bipartite PWS-IC in the maternal chromosome. It is known that the microdeletion of PWS-IC on the paternal chromosome result in loss of the expression of paternally expressed genes of both distal and proximal sites both in human and mice^15–18^. In contrast, the deletion of PWS-IC in the maternal chromosome does not alter the expression of the paternally expressed genes in the maternal chromosome. These results indicate that the DNA regulatory element of PWS-IC in the maternal chromosome is not directly implicated in silencing of imprinted genes in the maternal chromosome. A prevailing hypothesis is that maternal allele-specific DNA methylation in the PWS-IC silences expression of paternally expressed genes on the maternal chromosome^8^. However, supporting evidence for this hypothesis has been inconsistent. Loss of methylation of PWS-IC is observed in both mouse *Dnmt1*^−/−^ ESC and embryos. However, expression of *Snrpn* is mono-allelic in ESC but biallelic in embryos of *Dnmt1*^−/−20,49^. In contrast, expression of *Snrpn* is biallelic in *Ehmt2*^−/−^ ESC and embryos^19,27^. Interestingly, methylation of PWS-IC in the maternal chromosome is lost in ESC but intact in embryos of *Ehmt2*^−/−19^. These results indicate both DNMT1 and EHMT2 contribute to the methylation of PWS-IC in ESC but only DNMT1 is involved in the embryo. These are consistent with other reports that DNMT1 and EHMT2 interact directly^51,52^. This is also in line with the finding that different epigenetic machinery proteins are implicated in the epigenomic reprograming of demethylation during early development^53^. Consistent with these findings, we reported that EHMT2 inhibitors unsilence the paternally expressed *SNRPN/SNHG14* from the imprinted genes on maternal chromosome without changing the DNA methylation of PWS-IC^21^. Here, we showed that conditional inactivation of *Ehmt2* in the embryonic mouse brain reduces H3K9me2 levels without any changes in DNA methylation of the PWS-IC and unsilenced the expression of maternally imprinted *Snprn*. Unexpectedly, expression of the paternally imprinted genes *Magel2*, *Ndn*, and *Mkrn3* proximal to the centromere was not affected. These findings support that EHMT2 and H3K9me2 play a critical role in maintaining the silenced expression of PWS associated genes proximal to PWS-IC in the maternal allele. Surprisingly, our findings indicate that a distinct mechanism controls the silenced expression of paternally expressed genes in the distal side of PWS in the maternal chromosome.

Unique to this study, we were able to determine allelic specific chromatin accessibility, looping, and 3D chromatin folding of PWS imprinting domain because we employed the high-resolution epigenome and chromatin profiling technologies in human PWS and AS cell models with a paternal or maternal deletion respectively. The paternal allele is associated with a more open chromatin state and more chromatin loops while the maternal allele represents a more closed chromatin state and less loops. Notably, there is a scarcity of CTCF binding in the imprinted domain of 15q11.2-q13 compared to the 11p15.5 imprinted region associated with imprinted genes of *H19* and *IGF2*^42^. Through application of the newly developed high resolution chromatin tracing method, we were able to uncover an allele-specific 3D-folding organization of the PWS critical region with the 50kb genomic resolution.

We found that EHMT2 and H3K9me2 were enriched in the maternal PWS-IC. This enrichment was not affected upon inhibition of DNA methylation, suggesting that maternal DNA methylation is not required to maintain EHMT2 binding to the PWS-IC or instruct allele-specific EHMT2 binding. The transcript structures upstream and downstream of coding *SNRPN* exons are complex and have not been fully delineated. The tissue and transcript specific imprinted and non-imprinted expression pattern for these non-coding RNAs has been described^54,55^. Interestingly, we discovered that a new noncoding RNA (280118) associated with TSS4 is preferentially expressed from the maternal chromosome. The TSS4 and 280118 are distinct from the oocyte-specific u1A (TSS3) and u1B (TSS5) previously reported^45^. The exons of 280118 do not overlap with other adjacent transcripts that are predominately paternal or biallelic. These data suggest that 280118 transcript represent a distinct ncRNA and has a distinct function. Our RNA immunoprecipitation analysis supports that ncRNA 280118 that is preferentially expressed from the maternal chromosome plays a role in EHMT2 recruitment in CIS to the maternal PWS-IC. EMHT2 forms a local repressor or heterochromatin chromatin complex with SUV39H1 and HP1α and silences the expression of *SNRPN/SNHG14* on the maternal chromosome. It is noted that a similar mechanism has been described for the maternally expressed *Igf2r* gene: the paternally expressed antisense noncoding *Air* RNA mediates silencing of *Ig2r* expression in cis by recruiting EHMT2 to chromatin^25,56,57^. DNA elements within its locus are not required for *Air* RNA to silence distant imprinted genes. The same is observed for PWS-IC: deletion of PWS-IC DNA in the maternal chromosome does not affect the imprinted expression of paternally expressed genes^18^.

Our analysis did not reveal that EHMT2 inhibitor significantly enhances chromatin accessibility or changes the chromatin folding organization of the PWS imprinting domain using a global profiling method of ATAC-seq and targeted chromatin tracing method with 50kb genomic resolution. This is in contrast to the increased chromatin accessibility detected in a previous study using quantitative PCR of genomic DNA following in situ nuclease digestion^21^. It is possible that a change in chromatin accessibility after EHMT2 inhibitor treatment is at a micro-scale and can only be detected by epigenetic profiling methods with higher resolution. On the other hand, we discovered that EHMT2 inhibitor treatment in human fibroblasts significantly reduced the binding of maternal non-coding RNAs and EHMT2. The conformational change in EHMT2 is expected to lead to less formation of heterochromatin complex of EHMT2 with HP1α and SUV39H1 in the PWS-IC and unsilenced the expression of *SNPRN* from the maternal chromosome.

In summary, our study provides evidence supporting a new model of the mechanism of regulation of the PWS imprinted domain (Fig. 6h). The new model highlights the key discoveries from this study as well as incorporates exiting knowledge in the literature. First, the DNA methylation of PWS-IC serves in the establishment of imprinting but is not essential for imprinting maintenance. Second, our findings indicate a different mechanism operates to silence expression of paternally expressed genes distal or proximal to the PWS-IC on the maternal chromosome. This is in contrast with reports that PWS-IC likely employs the same mechanism to control the active expression of paternally expressed genes on the paternal chromosome at both distal and proximal sites. Third, we show for the first time that maternal non-coding RNAs upstream of PWS-IC interact with and recruit EHMT2 to PWS-IC, form a heterochromatin repressor complex, and silence the expression of *SNRPN/SNHG14* on the maternal chromosome. Thus, EMHT2 at PWS-IC plays a critical role in silencing expression on the maternal chromosome. Our findings have significantly advanced understanding of one of the most investigated imprinting domains to date.

## Materials and methods

### Human fibroblast cell culture

We obtained human fibroblasts from patients with PWS, AS and Ctr from the Baylor College of Medicine cell repository, Columbia University and Kansas University Hospital. We maintained human fibroblast cells in minimum essential medium alpha media (Gibco 12571–063) supplemented with 10% FBS (Gibco 10082–147), 1% l-glutamine (Gibco 25030–081), 100 units/ml penicillin and 100 μg/ml streptomycin (Gibco 15240–062) at 37 °C and 5% CO2 as previously described^21^.

### Animals

We handled all animals according to an Institutional Animal Care and Use Committee (IACUC) protocol approved by Yale University. Snrpn-EGFP mice^58^ were previously described. We obtained Ehmt2^f/+33^ mice from University of British Columbia and Nestin-Cre^+^ and C57BL/6J mice from the Jackson Laboratory. We used male and female mice in all studies.

### Co-immunoprecipitation

We washed human fibroblast cells cultured in a 10 cm dish with PBS and collected 5–6 million in 1.5 mL tube. After centrifugation at 4 °C for 5 min at 1500 rpm, we resuspended cell pellets by pipetting in 0.2 mL of non-SDS lysis buffer (150 mM NaCl, 50 mM Tris-HCl pH 7.4, 10% Glycerol, 1% Triton X-100) containing 1x protease/phosphatase inhibitor cocktail (Cell signaling). Cells were incubated on ice for 30 min and resuspended by pipetting every 10 min. After centrifugation at 4 °C for 30 min at 13,000 rpm, collected supernatants was quantified using Pierce^™^ BCA Protein Assay Kit (Thermo Fisher). The lysates (500 μg) were immunoprecipitated with 2 μg antibody (EHMT2, Invitrogen) or mouse IgG (Millipore) overnight at 4 °C with rotation. 30 μL of protein G-agarose beads (Roche) was added for two hour and then washed with HMTG buffer (20 mM HEPES pH 7.5, 150 mM NaCl, 0.1 % Triton X-100 and 10 % glycerol) containing 1x protease and phosphatase inhibitor three times at 4 °C for 5 min with rotation. The proteins were eluted in 2x Laemmli buffer (Bio-rad) by boiling at 98 °C for 5 min, and analyzed by immunoblotting.

### Chromatin immunoprecipitation

We used a Chromatin immunoprecipitation (ChIP) assay kit (Millipore) following the manufacturer’s instructions. Human fibroblast cells were fixed in 1% formaldehyde for 10 min at 37 °C, followed by two washes in cold PBS (Thermo scientific). Cells were scraped in PBS containing 1× protease/phosphatase inhibitor cocktail and resuspended in 0.2 mL SDS lysis buffer. Cells were incubated on ice for 20 min prior to lysing using a Bioruptor (Diagenode) for 12 cycles (10 sec on and 50 sec off), followed by centrifugation at 4 °C for 10 min at 14000 rpm. The sonicated cell supernatant was diluted in ChIP dilution buffer with protease inhibitor, 75 μL of Protein G Agarose (50% Slury) was added, after which the samples was incubated for 30 min at 4 °C with agitation. After brief centrifugation, the supernatant was collected and incubated with immunoprecipitating antibody (EHMT2, Invitrogen; H3K9me2, Abcam) overnight at 4 °C with rotation. Protein G Agarose was added for one hour and then washed with low salt, high salt, LiCl, and TE buffer for 5 min with rotation. To elute the precipitate, 250 μL of elution buffer was added and rotated at room temperature. After collecting the supernatant, the elution step was repeated. 20 μL 5M NaCl was added to combined elutes for reverse crosslinks at 65 °C for four hours, followed by added 10 μL of 0.5 M EDTA, 20 μL 1M Tris-HCl, and 2 μL of 10 mg/mL Proteinase K at 45 °C for one hour. DNA was recovered by phenol/chloroform (Sigma) extraction and precipitated by 40 μL 3M sodium acetate, 95% ethanol, and 20 μg glycogen. Pellets were washed with 70% ethanol and resuspended in double distilled water for qPCR reaction.

### RNA immunoprecipitation

We used a modified version of RNA immunoprecipitation protocol described by Raab et al ^59^. Human fibroblast cells were fixed in 0.3% methanol-free formaldehyde for 30 min at 4 °C, followed by quenching with 125 mM glycine for 5 min at room temperature. After three PBS washes, cells were collected in PBS containing 1 mM PMSF (phenylmethyl sulfonyl fluoride). After centrifugation, cell pellets were resuspended in 0.5 mL radioimmunoprecipitation assay (RIPA) buffer containing 0.5 mM Dithiothreitol (DTT, Thermo Scientific), 1× protease inhibitors (Thermo Scientific) and 2.5 μL RNAsin (Promega), followed by incubated on ice for 10 min prior to lysing using a Bioruptor (Diagenode) for four cycles of 5 sec on and 55 sec off. After centrifugation at 4 °C for 10 min at 14000 rpm, the supernatant was collected for incubation overnight at 4 °C with antibody-conjugated beads. Protein G magnetic beads (NEB) were pre-conjugated with EHMT2 monoclonal antibody (Invitrogen, A8620A) for 2 hours at 4 °C. Next day, beads were washed consecutively with fRIP buffer (25 mM Tris-HCl pH 7.5, 5 mM EDTA, 0.5% Ipegal CA-630, 150 mM KCl), followed by three times in ChIP buffer (50 mM Tris-HCl pH 7.5, 140 mM NaCl, 1 mM EDTA, 1 mM EGTA, 1% Triton X-100, 0.1% sodium deoxycholate, 0.1% SDS), one time in fRIP buffer for 5 min at 4 °C. After final wash, beads were resuspended in 3x reverse crosslinking buffer (3x PBS, 6% N-lauroyl sarcosine, 30 mM EDTA, 5 mM DTT). Eluted samples were collected to new tube and incubated with 20 μL proteinase K (Roche) and 1 μL RNAsin for one hour at 42 °C, one hour at 55 °C, and 30 min at 65 °C. RNA was extracted using Direct-Zol RNA miniprep kit (Zymo research) including the on-column DNase digestion. RNA was eluted in 12 μL double distilled water and used for qPCR reaction and library preparation.

### Bisulfite conversion sequencing

This experiment was performed using a EpiTect Bisulfite kit (Qiagen) following manufacturer’s instructions. In brief, isolated genomic DNA from human fibroblast or E18 mouse brain was treated with bisulfite and then 200 ng of input DNA was used for PCR amplification. We subcloned PCR products into pGEM-T easy vector (Promega), and we sequenced an average of 15 clones. We analyzed DNA-sequencing results using BISMA web-based analysis platform (http://services.ibc.uni-stuttgart.de/BDPC/BISMA/) with a setting for individual clones with <80% bisulfite conversion and <80% sequence identity to be excluded in the analysis. The primers that we used in this study are listed here. Human CpG 1’ (forward, 5′-ATTGTAATAGTGTTGTGGGGTTTTAGGG-3′; reverse, 5′-CCCAAACTATCTCTTAAAAAAAACCACC-3′), Human CpG 2’ (forward, 5′-TTTAAGTTTTTAGGATTTGGAGTATTGA-3′; reverse, 5′-AAACTACAATCACCCTAATATACCCAC-3′), Human CpG 3’ (forward, 5′-GGTGGGTATATTAGGGTGATTGTAGTTT-3′; reverse, 5′-CCTAATCCACTACCATAACCTCCTC-3′), and Mouse PWS-IC (forward, 5’-AATTTGTGTGATGTTTGTAATTATTTGG-3’; reverse, 5’-ATAAAATACACTTTCACTACTAAAATCC-3’)

### Western blotting

We performed western blotting as previously described ^60^. Whole cell lysates from human fibroblasts and mouse forebrains were prepared using 1x lysis buffer (Cell signaling) containing 1x protease/phosphatase inhibitor (Cell signaling). Histones were extracted using Core Histone Isolation kit (sigma) following manufacturer’s instruction. Samples were quantified by BCA assay (Thermo Fisher) and boiled in 4x Laemlli buffer (Bio-raad) at 98 °C for 5 min before loading in 4–20% precast gel (Bio-rad). The primary antibodies used to detect proteins are given in **Supplementary Table 1.**

### RT-PCR and qPCR

RNA was extracted from mouse forebrain of p11 or human fibroblasts using Trizol reagent (Sigma). cDNA synthesis was performed using a Reverse Transcription System kit (Promega). For quantitative real-time PCR (qPCR), PCR was performed on a CFX96 TouchTM Real-Time PCR Detection System (Bio-Rad). The primers used to amplify cDNAs are given in **Supplementary Table 1.** Ct values for each sample were obtained using CFX Manager Software version 3.0 (Bio-Rad).

### Chromatin associated RNA (chrRNA) fractionation

We performed chrRNA using a protocol described in Sledziowska et al ^47^. Human fibroblasts were treated with 1ml of TrypLE(Gibco) per well in a 6-well plate. The cells were incubated until they detached, at which point 2 ml of DPBS was added per well. The cells were collected and centrifuged at 200 g for 5 min. The supernatant was removed and the cells were resuspended in the 200 μl of cytoplasmic lysis Buffer (0.15% NP-40, 10mM Tris–HCl pH 7, 150mM NaCl, 50U RiboLock). After incubating samples for 5 min on ice, they were layered on 500 μl of sucrose buffer (10mM Tris–HCl pH 7, 150mM NaCl, 25% Sucrose, 50U RiboLock). Nuclei were collected by centrifugation of 16000 g for 10 min at 4°C. The supernatant containing cytoplasmic fraction was then removed and nuclei were washed with nuclei wash buffer (1X PBS supplemented 0.1% TritonX-100, 1mM EDTA, 50U RiboLock) at 1200 g for 1 min at 4°C. Supernatant was discarded and the nuclei were resuspended in 200μl of glycerol buffer (20mM Tris–HCl pH 8, 75mM NaCl, 0.5mM EDTA, 50% glycerol, 0.85mM DTT, 50U RiboLock). 200μl of nuclei lysis buffer (1% NP-40, 20mM HEPES pH7.5, 300mM NaCl, 1M Urea, 0.2mM EDTA, 1mM DTT, 50U RiboLock) was added, following 2 min of vortexing by pulsed. After centrifuged at 18500 g for 2 min at 4°C, the pellet containing chrRNA was resuspended in 200μl of PBS supplemented with 50U RiboLock. Following resuspension, RNA was extracted using Trizol reagent for library prep.

### RNA-seq

All procedures were conducted in the Yale Center for Genome Analysis.

RNA Seq Quality Control: total RNA quality was determined by estimating the A260/A280 and A260/A230 ratios by nanodrop. RNA integrity was determined by running an Agilent Bioanalyzer gel, which measures the ratio of the ribosomal peaks. For library prep, we used samples with RIN values of 5 or greater.

RNA Seq Library Prep: for mouse forebrain samples, using the Kapa RNA HyperPrep Kit with RiboErase (KR1351), rRNA is depleted starting from 25–1000ng of total RNA by hybridization of rRNA to complementary DNA oligonucleotides, followed by treatment with RNase H and DNase to remove rRNA duplexed to DNA. Samples are then fragmented using heat and magnesium. 1^st^ strand synthesis is performed using random priming. 2^nd^ strand synthesis incorporates dUTPs into the 2^nd^ strand cDNA. Adapters are then ligated and the library is amplified. Strands marked with dUTPs are not amplified allowing for strand-specific sequencing. Indexed libraries that meet appropriate cut-offs for both quantity and quality are quantified by qRT-PCR using a commercially available kit (KAPA Biosystems) and insert size distribution determined with the LabChip GX or Agilent Bioanalyzer. Samples with a yield of ≥0.5 ng/ul are used for sequencing. For human fibroblast samples, using the SMARTer Stranded Total RNA-Seq Kit v3-Pico Input Mammalian from Takara and a normalized RNA input between 250pg-10ng, the RNA is first fragmented prior to first strand cDNA synthesis. Next indexing and PCR1 occurs. After a bead clean-up, ribosomal cDNA is depleted by ZapR v3 in the presence of mammalian specific R-probes. Next, fragments that are not cleaved in the depletion step are enriched in a second PCR before a final bead clean-up is performed. Indexed libraries that meet appropriate cut-offs for both quantity and quality are quantified by qRT-PCR using a commercially available kit (KAPA Biosystems) and insert size distribution determined with the LabChip GX or Agilent Bioanalyzer. Samples with a yield of ≥0.5 ng/ul are used for sequencing.

Flow Cell Preparation and Sequencing: sample concentrations are normalized to 1.2 nM and loaded onto an Illumina NovaSeq flow cell at a concentration that yields 25 million (human fibroblast RNA) or 50 million (mouse forebrain RNA) passing filter clusters per sample. Samples are sequenced using 100bp paired-end sequencing on an Illumina NovaSeq according to Illumina protocols. The 10bp unique dual index is read during additional sequencing reads that automatically follow the completion of read 1. Data generated during sequencing runs are simultaneously transferred to the YCGA high-performance computing cluster. A positive control (prepared bacteriophage Phi X library) provided by Illumina is spiked into every lane at a concentration of 0.3% to monitor sequencing quality in real time.

Data Analysis: signal intensities are converted to individual base calls during a run using the system’s Real Time Analysis (RTA) software. Base calls are transferred from the machine’s dedicated personal computer to the Yale High Performance Computing cluster via a 1 Gigabit network mount for downstream analysis. Primary analysis - sample de-multiplexing and alignment to the mouse genome - is performed using Illumina’s CASAVA 1.8.2 software suite. The sample error rate is less than 2% and the distribution of reads per sample in a lane is within reasonable tolerance.

### RNA-seq data analysis and Gene ontology analysis

Reads were mapped to the mouse genome (mm10) using STAR software ^61^. DESeq2 ^62^ software was applied to the counts of protein coding genes to estimate the fold-change between the samples from mice that Ehmt2^f/f^;p^S-E^/m^+^ versus Nestin-cre^+/−^;Ehmt2^f/f^;m^S-E^/p^+^. Analyses in gene set enrichment analysis (v4.1.0) were performed on normalized counts generated in DESeq2 to analyze whether published gene sets were significantly enriched in either the Ehmt2^f/f^;p^S-E^/m^+^ or Nestin-cre^+/−^;Ehmt2^f/f^;m^S-E^/p^+ 63^. The analysis used c5.all.v.7.4.symbols.gmt (gene ontology) gene set databases with default parameters. A false discovery rate (FDR) of less than 25% was used as a cut-off for a gene set to be significantly enriched. Gene expression levels were converted into heatmaps and colors quantitatively correspond to fold-changes. For analysis of chrRNA-seq and bulk RNA-seq, reads were mapped to the human genome (hg38) by Burrows-Wheeler Aligner (v0.7.12). The alignment BAM files were converted to bigWig file format and visualized in the UCSC Genome Browser (https://genome.ucsc.edu/) ^64^.

### ATAC-seq

ATAC-seq libraries were constructed with 5×10^4^ human fibroblast cells following Omni-ATAC protocol (Illumina FC-121–1031) ^65^. The libraries were sequenced on Illumina Nextseq 500 (paired-end run, 42 bp): sequenced reads were trimmed with adaptor sequences (cutadapt v1.9.1) ^66^ and mapped to the human genome (GRCh38/hg38) by Bowtie2 (v2.3.4.1) ^67^. Mitochondrial and duplicated reads were removed by SAMtools (v1.9) ^68^ and Picard (v2.9.0, https://broadinstitute.github.io/picard/), respectively. Peaks were found by MACS2 (v.2.1.1) ^69^ and visualized by deepTools (v3.1.1) ^70^. Motif enrichment analysis of ATAC-seq peaks was done by HOMER (v4.10) ^71^. P-values for motif enrichment were calculated using cumulative binomial distribution.

### DNA methylation array

The Infinium MethylationEPIC Kit (Illumina) was used to measure DNA methylation profiles from the eight human fibroblast lines. Genomic DNA was extracted using DNeasy blood & tissue kit (Qiagen) following the manufacturer’s instruction. DNA input of 500 ng was preprocessed for bisulfite conversion and DNA methylation profiling was conducted at Yale Center for Genome Analysis as previously described ^72^. GenomeStudio software (Illumina) was used for Methylation EPIC data analysis.

### Hi-C and bioinformatics analysis

Hi-C data was generated using the Arima-HiC kit (Arima Genomics, A510008), according to the manufacturers protocols and analyzed at Yale center for Genome Analysis. Briefly, human fibroblasts grown on 100 mm-diameter dishes were collected and resuspended in media for crosslinking with formaldehyde (the final concentration; 2%). After incubation at room temperature for 10 min, stop solution 1 was added and incubated at room temperature for 5 min. We then followed the instruction described in User Guide (Document# A160134 v01) to purify DNA for library preparation. Before proceeding to library preparation, we did shallow sequencing for quality determination following the Arima-HiC QC Quality Control protocol (**Supplementary Table 2**). For studying 3D genome conformation, we obtained 700 million read-pairs per sample. HiC library was sequenced in paired-end mode (2×150bp read length) with NovaSeq (Illumina) and mapped to human genome hg38. For analysis, we used Juicer providing a pipeline from processing raw Fastq reads to high-order analysis including contact domain and chromatin loops ^73^. To visualize .hic.file, we used Juicebox ^74^, WashU Epigenome Browser ^75^, and IGV ^76^

### Probe design and synthesis for chromatin tracing

To design DNA FISH probes for chromatin tracing, the genomic regions of interest (Chr15: 23,500,000–25,650,000, hg38) were each divided into 42 consecutive 50-kb target segments. For each 50-kb target segment, 500 oligonucleotides were designed as template oligos. On each template oligo, the following sequences were concatenated from 5′ to 3′: (1) a 20-nucleotide (nt) forward priming sequence, (2) a 20-nt secondary probe binding sequence, (3) a 30-nt genome targeting sequence, (4, 5) two 20-nt secondary probe binding sequences, and (6) a 20-nt reverse priming sequence. The 30-nt genome targeting sequences were designed with by ProbeDealer ^77^ with an extra BLAST ^78^ against the repetitive genome to remove repetitive target sequences. The template oligo pool for primary probes was purchased from TWIST Bioscience. The probes were synthesized as described previously ^43,79–81^.

### Primary probe hybridization for chromatin tracing

The experiment was modified from our published protocol ^43,79–81^. The human fibroblasts derived from patients grown on a 40-mm-diameter coverslip were fixed in 4% (w/v) paraformaldehyde for 10 min at room temperature, followed by twice DPBS washes. Cells were then permeabilized with 0.5% (v/v) Triton X-100 in 1x DPBS for 10 min at room temperature, followed by two DPBS washes. Fibroblasts were treated with 0.1 M HCl for 5 min at room temperature and washed twice with DPBS, followed by a 45 min treatment with ribonuclease A (RNase A) (0.1 mg/ml) in DPBS at 37°C and two DPBS washed. Cells were then incubated with prehybridization buffer composed of 50% (v/v) formamide and 0.1% v/v Tween-20 in 2x saline sodium citrate (SSC) for 30 min at room temperature. After carefully removing excess liquid by dipping on tissue paper, the coverslip was flipped onto a glass slide and contacted with 30 ul hybridization buffer composed of 50% (v/v) formamide, 20% (w/v) dextran sulfate and 20 uM primary probes in 2x SSC. The samples were then heat-denatured on an 86°C heat block (with a surface temperature of ~80°C) for 3 minutes and incubated in a humid chamber overnight (>18 hours) at 37°C. Next, the samples were then washed with 0.1% (v/v) Tween 20 in 2x SSC in a 60°C water bath twice for 15 min each and once at room temperature for 15 min.

### Sequential hybridization of secondary probes for chromatin training

After primary probe hybridization, the coverslip was assembled into a Bioptech’s FCS2 flow chamber and connected to a homebuilt automated fluidics system ^43,79^. To read out each genomic locus, we used adapter oligos and common readout oligos that were labeled with Cy3 or Cy5 dye through a disulfide bond to enable signal removal by tris(2-carboxyethyl)phosphine (TCEP) wash. Each adapter oligo is composed of one 20-nt primary probe binding sequence (that binds to the overhangs on primary probes) and two replicates of the same 20-nt common readout oligo binding sequences (**Supplementary Table 3**). Before each round of imaging, the sample were incubated with secondary hybridization buffer composed of 20% (v/v) ethylene carbonate (EC), two 10nM adapter oligos and two 15nM Cy3- and Cy5-labeled common readout oligos in 2x SSC. To perform the imaging, we took z-stepping images with 647-nm, 560-nm, and 488-nm laser illuminations for Cy5 and Cy3 readout oligos and fiducial beads respectively, with 200-nm step sizes and 0.4-s exposure time at each step. After each round of imaging, the signals were removed by TCEP washing buffer composed of 20% (v/v) EC, 50mM TCEP and 1uM blocking oligos in 2xSSC. Blocking oligos are dye-free common readout oligos to block any unoccupied binding sites. This procedure was repeated 21 rounds until all 42 segments were imaged.

### Chromatin tracing analysis

Before foci fitting, the color shift between the 560-nm and 647-nm laser channels were corrected with TetraSpeck bead images; the sample drift during sequential hybridization and imaging were corrected with fiducial bead images. Next, cell nucleus was segmentation with DAPI images and used as a mask to fit the 3D positions of loci only inside the cell nucleus. The fitted loci were then linked into chromatin traces based on their spatial clustering patterns. Finally, we tried to re-fit the missing loci in chromatin traces within the chromatin trace region in corresponding hybridization and added them to the chromatin traces.

### Availability of data and materials

Previously published data were downloaded from ^27^, ^82^.

### Statistical analysis

We used Graphpad Prism for the statistical analyses. Differences between groups were analyzed by one-way ANOVA followed by Dunnett’s multiple comparison test or two-way ANOVA followed by Šídá’s multiple comparisons test or unpaired two-tailed Student’s *t* test, where appropriate. ^*^p < 0.05 was considered statistically significant (^**^p < 0.01, ^***^p < 0.001, ^****^p < 0.0001).

## Extended Data
